# Embedding telerehabilitation in practice: Adapting NeuroRehabilitation OnLine (NROL) for a new context through co-production and implementation science knowledge

**DOI:** 10.1177/20552076261484770

**Published:** 2026-09-18

**Authors:** Suzanne Ackerley, Amy Bastow, Ruth Witham, Louise Jackson, Adam Partington, Helen Vernon, Louise Connell

**Affiliations:** 1 Faculty of Health & Medicine, Health Innovation Campus, 4396 Lancaster University, Lancaster, UK; 2 Rakehead Rehabilitation Centre, Burnley General Teaching Hospital, 8943 East Lancashire Hospitals NHS Trust, Burnley, UK; 3 Cheshire West and Chester Integrated Community Stroke and Neuro Service, Countess of Chester and Cheshire and Wirral Partnership NHS Trust, Chester, UK; 4 Cheshire and Mersey Integrated Stroke Delivery Network, Cheshire and Mersey, UK; 5 Adult Speech and Language Therapy Team, Countess of Chester NHS Foundation Trust, 2387 Countess of Chester Hospital, Chester, UK; 6 Liverpool and South Sefton Early Supported Discharge Team, 4595 University Hospitals Liverpool Group, Liverpool, UK

**Keywords:** adaptation, scalability, ADAPT guidance, TIDieR-Rehab checklist, ISAT

## Abstract

**Background:**

Delivering recommended intensity of neurorehabilitation remains a challenge, with telerehabilitation offering one solution. NeuroRehabilitation OnLine (NROL) is a multidisciplinary, group-based telerehabilitation model embedded within one region in England, showing promise and alignment with healthcare strategic priorities. The importance of scaling successful evidence-informed practices is recognised, however careful adaptation is required to ensure contextual fit and sustainability.

**Objective:**

To describe the adaptation of NROL for implementation in a new context.

**Methods:**

The four-step ADAPT guidance was applied with previously identified implementation strategies to guide the adaptation of NROL from Region 1 to Region 2. Adaptation activities were co-produced. Contextual factors were detailed using the Consolidated Framework for Implementation Research and the Intervention Sustainability Assessment Tool. The adapted innovation was described using the TIDieR-Rehab checklist.

**Results:**

NROL was successfully adapted for Region 2. Step 1 confirmed strategic fit but identified barriers including workforce, infrastructure and resource. Led by an adaptation team and supported by a learning collaborative, Step 2 responded to barriers, retaining core components while tailoring role configuration and materials. Step 3 demonstrated feasibility and acceptability through piloting and phased integration, and improved fit within service pathways. Step 4 focused on sustainment, supported by training, stakeholder engagement, and reporting.

**Conclusion:**

This study offers key considerations for scale-up, providing an example of context-sensitive adaptation to a new region, demonstrating how frameworks and co-production can support the adaptation of telerehabilitation models. This example has potential wider use for researchers and implementers tasked with delivering impact, though highlights the effort and resource needed.

## Background

Delivering the recommended intensity of neurorehabilitation remains a persistent global challenge, with workforce shortages, geographic disparities, and fragmented care pathways.^1–3^ Telerehabilitation may help address these gaps, increasing access to specialist rehabilitation through flexible, remote delivery.^4^ In stroke and other neurological populations, telerehabilitation has been used to deliver allied health interventions.^5–7^ Evidence from systematic reviews indicates it can be delivered safely,^8^ and can yield outcomes comparable to conventional in-person therapy, including for activities of daily living and health-related quality of life.^5–7^ Telerehabilitation has been endorsed in international stroke clinical guidelines for use alongside conventional in-person therapy.^9–12^

NeuroRehabilitation OnLine (NROL) is an innovation that provides real-time, multidisciplinary, group-based, telerehabilitation with dedicated technology assistance.^13^ It combines targeted therapy with peer-support aiming to enhance access and opportunity for neurorehabilitation while making efficient use of the workforce. Recent publications provide a comprehensive description of NROL’s components,^13^ and successful integration within the National Health Service (NHS) stroke and neurorehabilitation services in one region of England.^14,15^ These highlight NROL’s alignment with national guidelines,^9,10^ healthcare strategic priorities,^16–18^ and its adaptability to local contexts.

Scaling evidence-informed practices, like NROL, is increasingly recognised as a strategic objective for health systems. The World Health Organization reports scale-up as “the deliberate effort to increase the impact of successfully tested health interventions so as to benefit more people and to foster policy and program development on a lasting basis”.^19^ Adaptation is a key part of successful scale-up, with careful attention required to how well an intervention will fit and function in the new context, including considering infrastructure, workforce, readiness, patient needs and sustainment. Despite the importance of reporting intervention adaptations, a recent review found many studies lack description in full.^20^ The ADAPT guidance was developed to provide a systematic, stepwise approach to support structured and transparent adaptation.^21^ Alongside ADAPT, TIDieR checklists provide structured tools for describing interventions in sufficient detail to support replication and fidelity.^22–24^ Despite their availability, to the authors knowledge no studies have formally applied both ADAPT and TIDieR comprehensively to describe innovation adaptation in the context of telehealth.

Combining complementary implementation science knowledge with the ADAPT guidance can help navigate the complexities of transferring interventions across diverse health systems.^21,25^ While ADAPT provides a structured process for modifying interventions, it relies on a clear understanding of context to determine what needs to change and why. The widely applied Consolidated Framework for Implementation Research (CFIR) supports systematic identification of contextual determinants.^26^ The value of concurrent use of the CFIR to operationalise ADAPT guidance was highlighted by Connell et al. (2025), using NROL as an example.^27^ They used the ADAPT guidance and CFIR, in conjunction with the updated Medical Research Council framework for the development and evaluation of complex interventions,^28^ to develop, adapt, implement and evaluate NROL as a scalable regional innovation. Of note, incorporation of the CFIR enabled identification of Expert Recommendations for Implementing Change (ERIC) implementation strategies^29^ (through the CFIR-ERIC matching tool) to support implementation efforts, including for the adaptation phase.

Building on prior work, this paper focuses on adapting NROL for delivery in a new context, a second NHS region within England. Using the ADAPT guidance and TIDieR-Rehab checklist, the adaptation process is documented along with contextual considerations and applied implementation strategies. This publication aims to offer considerations for therapy staff, service leads, and digital health implementers seeking to adapt or scale telehealth models.

## Methods

The process was iterative and grounded in co-production principles, with the adaptation of NROL applying complementary implementation science knowledge.

This work was undertaken as a service development project, focusing on adapting and implementing an existing model of care. Reporting covers a period of 24 months (April 2023 to March 2025), from pre-implementation through to implementation of three full 6-week NROL blocks in Region 2. No individual-level research data were collected. Stakeholder contributions formed part of co-production activities for service design and implementation. Descriptions are presented at the service or group level, and individual contributors are not identified. In line with Health Research Authority guidance, formal research ethics approval was not required as the activities constituted service development rather than research.^30^

ADAPT guidance provided the procedural structure defining *how* adaptation was undertaken^21^; the CFIR and Intervention Scalability Assessment Tool (ISAT) guided contextual enquiry and informed *what* required adaptation by identifying barriers, facilitators, and readiness within the new context^26,31^; and selected ERIC strategies organised stakeholder engagement.^29^ This complementary use of implementation science conceptual knowledge enabled a structured yet context-sensitive approach to adaptation, with CFIR-informed insights guiding decisions within each ADAPT step.

A pre-print of this manuscript was made available online.^32^

### Stakeholder co-production

A key feature of NROL’s development, implementation and adaptation has been co-production, with key stakeholders (including therapy staff, service leads, patients, clinical-academics, and members of the stroke and neurorehabilitation community) actively shaping the innovation. This collaborative model supports contextual relevance, clinician buy-in, and patient engagement; factors known to influence implementation success and sustainability.^33^

For this project, a coalition was built between Region 2 (Cheshire and Merseyside) and Region 1 (Lancashire and South Cumbria), facilitated by established connectivity between their regional stroke networks and a shared purpose to improve neurorehabilitation provision, with NROL identified as one possible solution. The foundational adaptation work was led by coalition members; designated Regional Stroke Rehab Leads from Region 2 and NROL Leads from Region 1 (n=5). Subsequently, an NROL adaptation team was formed (n=12), comprising the Leads from the coalition (as above) and the NROL operational teams from both regions. Further, a regional NROL learning collaborative was established, including existing workforce, patients, and members of the stroke community, and was iterated over time. Details on the stakeholder groups, including membership and roles are provided in Supplemental file 1. The adaptation of NROL for Region 2 was shaped by co-production principles emphasising shared power and mutual respect.^34,35^ Individuals contributed their perspectives and skills with all forms of knowledge valued equally. Fostering reciprocal relationships, prioritising relationship-building and maintaining open communication, ensured the project met the needs of all stakeholders.

#### Positionality statement

The author team comprises clinical-academic staff, and operational, service and regional leads, embedded within the stroke and neurorehabilitation services across both regions involved in this study. Our direct involvement in the development, delivery, and adaptation of NROL provided deep contextual insight and facilitated co-production. We acknowledge that our dual roles, as implementers and evaluators, may have influenced interpretation. To mitigate bias, diverse stakeholders were engaged throughout and structured frameworks used to guide and document the process.

### Process for adapting NROL

Adaptation of NROL in Region 2 used a multi-tool approach, with selection of tools informed by complementary strengths in structuring adaptation processes; capturing contextual influences, ensuring transparent reporting, and fit within the larger programme of work previously described.^27^

The ADAPT guidance was the overarching tool, offering a stepwise approach to modifying the innovation for a new context.^21^ NROL was adapted informed by four key steps, as outlined in Figure 1. This guidance was particularly suited to the current study, which aimed to scale-up an existing innovation to a new region while maintaining fidelity to its core components. Adaptation was supported using previously identified ERIC implementation strategies.^27^ ERIC strategies help inform actions to support effective implementation.^29^ Strategies focused on stakeholder interrelationships, training and education, and evaluation and iteration.^36^

**Figure 1.**
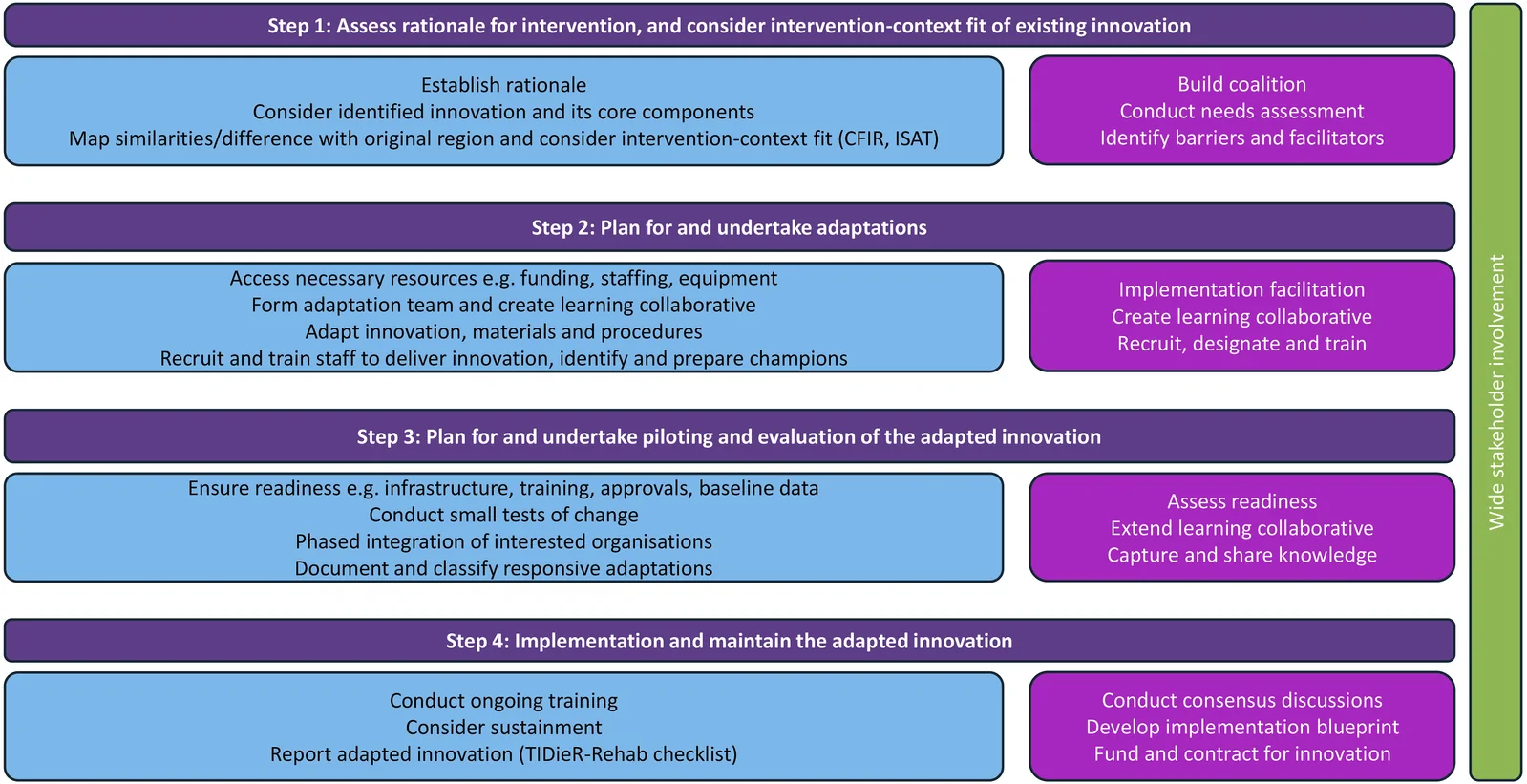
Process for adapting NeuroRehabilitation OnLine (NROL) to a new context, informed by the ADAPT guidance,^21^ Consolidated Framework for Implementation Research (CFIR),^26^ Intervention Scalability Assessment Tool (ISAT),^31^ and reported with the TIDieR-Rehab checklist.^23^ Purple boxes = Steps of the ADAPT guidance.^21^ Blue boxes = Expected outcomes from each step. Pink boxes = ERIC implementation strategies applied.^27^

In Step 1, the CFIR and ISAT supported contextual analysis. The CFIR was used to operationalise ADAPT guidance, offering a comprehensive lens to systematically detail and compare contextual factors likely to influence implementation (innovation characteristics, outer setting, inner setting, characteristics of individuals) across regions.^26^ Barriers and facilitators to NROL implementation in Region 2 were identified by NROL coalition members during regional network meetings, and shared by Region 1 NROL Leads drawing on experience and prior evaluation. These were subsequently synthesised under CFIR domain headings by NROL Leads. Barriers were factors likely to inhibit the integration of NROL into practice, whereas facilitators were those that should enable or promote integration. It was recognised that some factors could function as both over time (e.g. time-limited resources). The ISAT provided a structured assessment of context-specific factors influencing readiness for scale-up, covering two parts: A-Background contextual information and B-Implementation and sustainability considerations (see Figure 2 for Domain headings).^31^ Domains were scored between 0 (not at all) and 3 (to a large extent) collaboratively. Initial scoring was completed by two members of the adaptation team, with final scores agreed by consensus with the wider team. Scores were summarised via radar plot.

**Figure 2.**
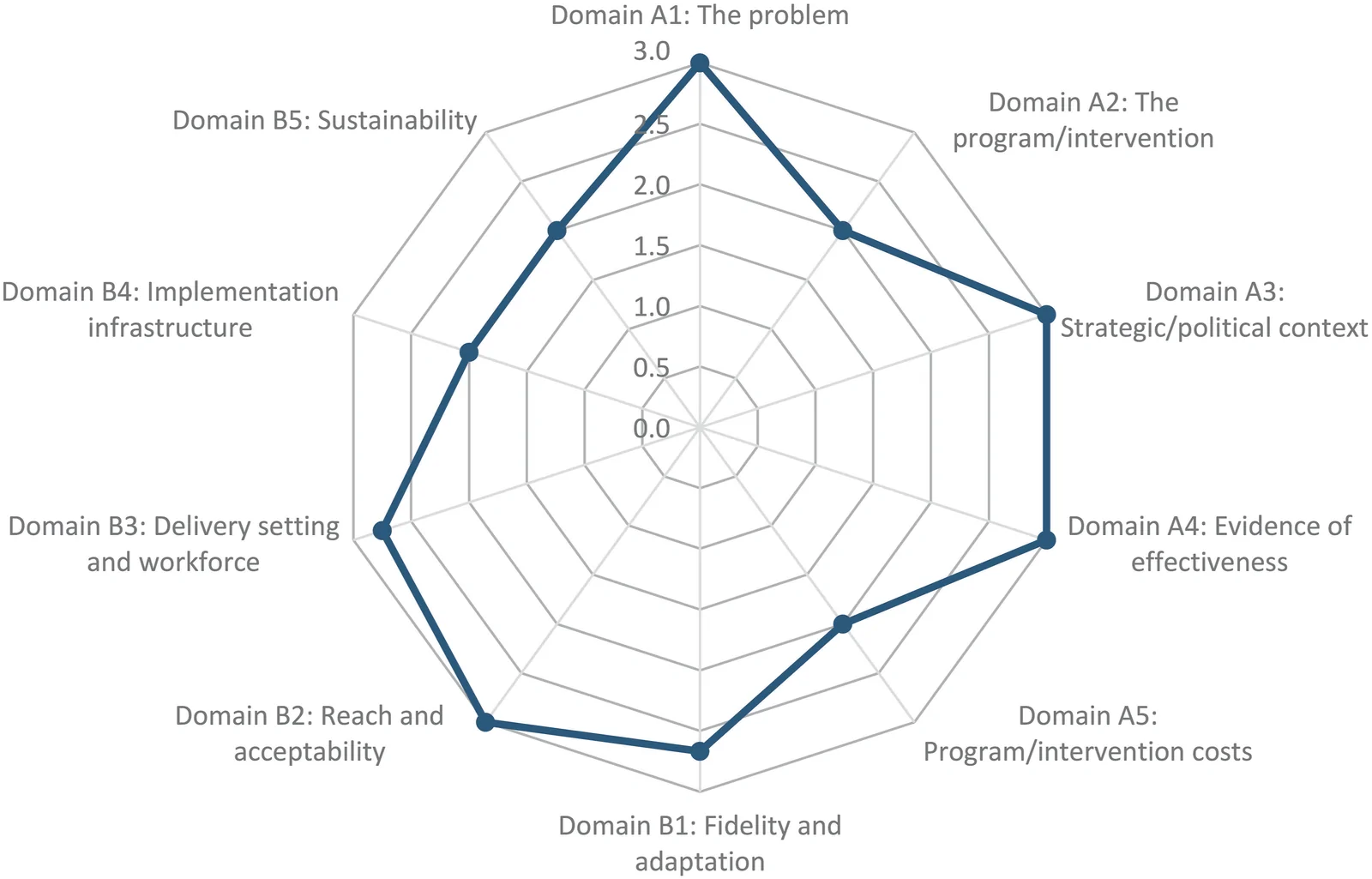
Intervention Scalability Assessment Tool (ISAT)^31^ radar plot depicting factors influencing Region 2’s readiness for NROL implementation. Domains A1-5 focus on background contextual information and B1-5 on implementation and sustainability. Each domain is scored for readiness between 0 (not at all) to 3 (to a large extent), therefore facilitators are depicted nearer the outside of the circle and barriers more centrally.

In Step 4, the TIDieR-Rehab checklist,^23^ with the Telehealth extension,^24^ was used to report the adapted innovation. The TIDieR-Rehab extends the original TIDieR checklist^22^ to better capture the complexity of rehabilitation interventions. Initial drafting was undertaken by the authors and refined through consultation with Region 2 learning collaborative members. Content for the TIDieR-Rehab checklist was systematically updated from Region 1’s TIDieR checklist,^13^ with region specific content (new/adapted/removed) clearly marked.

## Results

### Step 1: Assess rationale for intervention, and consider intervention-context fit of existing innovation

#### Establish rationale, and consider identified innovation and its core components

Region 2 faced persistent challenges in delivering the recommended levels of stroke rehabilitation, compounded by workforce shortages and fragmented pathways, and anticipated increased prevalence.^37^ Regional Stroke Rehab Leads from the region’s stroke delivery network identified NROL as one promising solution, informed by exposure at meetings, conferences and in literature.

#### Map similarities/difference with original region and consider intervention-context fit

Table 1 shows the comparison of the similarities and differences between Region 1 (original) and Region 2 (new) and identifies the barriers and facilitators to NROL implementation.

**Table 1.** Comparison of contextual factors between original and new regions, including barriers and facilitators to NROL implementation.

​	Region 1 (original region)	Region 2 (new region)
*Innovation*	- NROL at **Lancashire and South Cumbria**	- NROL at **Cheshire and Merseyside**
*Outer setting (External influences)*	Aligns with healthcare system strategic priorities^16,17^ (F) Ambition to increase therapy provision toward National Clinical Guideline targets^9,10^	
*Inner setting (Regional setting)*	- **1.8 million people** - Urban, rural and coastal areas - Areas of low deprivation and neighbourhoods with lower than national average healthy life expectancy	- **2.7 million people** - Urban, rural and coastal areas - Areas of low deprivation and neighbourhoods with lower than national average healthy life expectancy
*Inner setting (Healthcare setting)*	- **4 organisations, 7 community stroke and/or neurorehabilitation teams involved** - Difficulty achieving guideline recommended amounts of therapy, ambition for equitable access to more rehabilitation - **Initiated within an established clinical-academic partnership (ELHT/LU) (F)** - **NROL champions identified over time (F)** - Leadership commitment (F) - Dedicated NROL roles (F) - Limited workforce and resource (B) - Varied service- and infra- structure across organisations, and constantly changing (B) - **Fixed-term funding (F/B)**	- **10 organisations, 18 community and/or early supported discharge stroke teams interested in involvement** - Difficulty achieving guideline recommended amounts of therapy, ambition for equitable access to more rehabilitation - **Initiated by Regional Stroke Rehab Leads (F)** - Aware upfront effort required for implementation (B) - **Recent regional technology project, with members identified as potential champions for NROL (F)** - Leadership commitment (F) - **Strong clinician interest from prior NROL dissemination (F)** - Need for dedicated NROL roles (B) - Limited workforce and resource (B) - Varied service- and infra- structure across organisations, and constantly changing (B) - **Need for funding (B)**
*Individuals (roles)*	- Patients: Active service-users (≥18 years) with stroke or other neurological condition - Staff: NROL operational team (Lead, **Technology Support, Assistant,** Administrator), existing NHS therapy workforce across integrated organisations - **Clinical-academic (ELHT/LU) and charity (SameYou) partners (F)** - Members of the stroke and neurorehabilitation community (**Ex-NROL volunteer**, **Stroke Association Support Coordinators & stroke survivors**) and student involvement (F)	- Patients: Active service-users (≥18 years) with **stroke** - Staff: Need for NROL operational team (Lead, **Technology Support (x2)**, Administrator), existing NHS therapy workforce across integrated organisations - **No established clinical-academic partnership (B)** - Potential for members of the stroke community (**Stroke Association Support Coordinators**) and student involvement (F)

Bold, difference between regions. (F) = Facilitator, (B) = Barrier, (B/F) = Both facilitator and barrier (i.e. fixed-term funding supported project initiation but its time-limited nature hinders sustainment). NROL = NeuroRehabilitation OnLine, ELHT = East Lancashire Hospitals NHS Trust, LU = Lancaster University, NHS = National Health Service.

Key differences included that Region 2 was larger (population and number of organisations) and had a stroke-specific focus, with future ambition to extend to other neurological conditions. While most eligible organisations (10/14) showed interest in NROL, four opted not to participate due to competing priorities or a current hesitancy toward integrating new initiatives and technology. Another difference was the absence of an established clinical-academic partnership for the project, contrasting Region 1 where such a partnership was central. Region 2, however, felt they benefitted from Regional Stroke Rehab Lead initiation and leadership, as they had existing knowledge of the ‘people and processes’ covering all organisations and recent technology project engagement, which could help expedite upfront implementation efforts (e.g. stakeholder engagement, governance approvals).

Key similarities included recognition that NROL had strong strategic alignment,^16,17^ a significant facilitator. Both regions identified limited workforce and resource, and varied service- and infra-structure across organisations as barriers for implementation. There was a preference to maintain dedicated NROL operational roles, however funding was needed to recruit staff. Two Technology Support roles were prioritised due to existing availability of therapy assistance within the workforce and the size of the region. Region 2 recognised NROL’s potential to optimise workforce use and drew on Region 1’s solutions to service challenges.

Figure 2 depicts the factors influencing Region 2’s context-specific readiness for NROL implementation. ISAT scoring showed NROL was seen as a valuable innovation, though not a panacea (A2). Funding needs (A5), infrastructure gaps (B4) and long-term sustainability (B5) were identified as areas for response, notably highlighting need for dedicated NROL operational staff.

### Step 2: Plan for and undertake adaptations

#### Accessing necessary resource

In response to the identified barriers, seed funding was secured through a national health funding body enabling recruitment of the dedicated NROL operational team and a modest budget for equipment and materials.

#### Form adaptation team and create learning collaborative

An adaptation team was formed, and met regularly to support iterative, principled adaptation of NROL for Region 2. Adaptations were informed by Step 1, ongoing regional, organisational and service needs, and stakeholder feedback. The new region viewed the involvement of the original region positively. A regional NROL learning collaborative was established, and was iterated over time, to support shared decision-making and co-production. Examples of co-production activities and contributions are provided in Supplemental file 1.

#### Adapt innovation, materials and procedures

Adaptations were planned and undertaken to achieve the best fit of the NROL core and peripheral components within Region 2, and key components are summarised in Figure 3. The remaining peripheral components were all maintained and are described in the TIDieR-Rehab checklist (Supplemental file 2). Responsive adaptations are detailed in Step 3.

**Figure 3.**
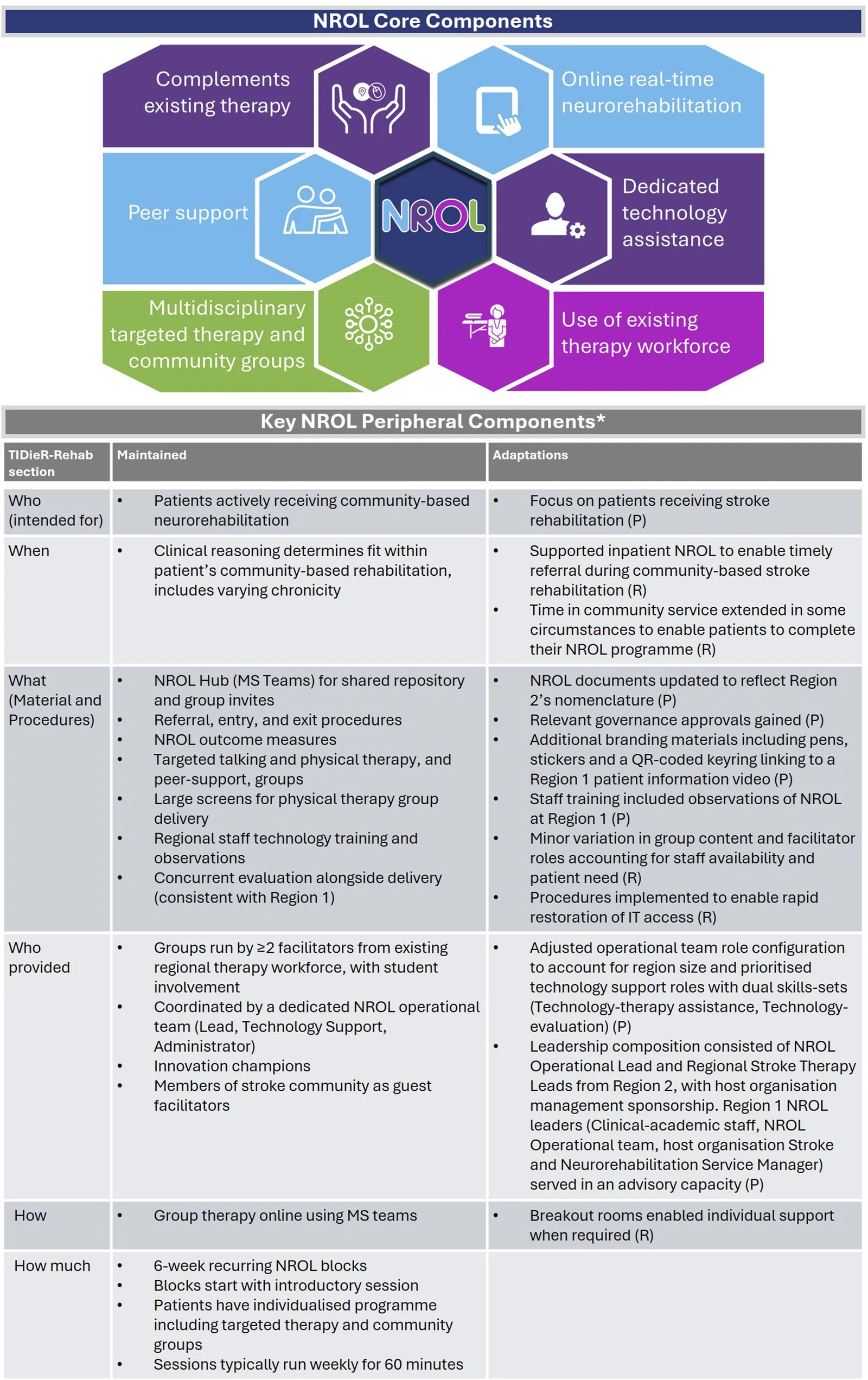
Core and key peripheral components for NROL adapted for region 2. Key peripheral components are detailed under TIDieR Rehab sections. Planned adaptations (P) determined during ADAPT step 2 and responsive adaptations (R) during ADAPT step 3. *Remaining peripheral components were maintained and are detailed in the TIDieR-Rehab checklist (Supplemental file 2).

All NROL core components were retained. The description of one core component was modified to clarify the model was intended for use in ‘neurorehabilitation’ rather than specifying stroke and neuro-rehabilitation.

Key NROL peripheral components were aligned with the relevant TIDieR-Rehab section headings and categorised as either ‘maintained’ or ‘adaptations’. The basic structure of NROL was maintained (i.e. How/How much). Planned adaptations were made to the ‘Who’ NROL was intended for and ‘Who provided’ and included offering NROL only to patients receiving stroke rehabilitation, and adjustment to operational team and leadership role configuration. Planned adaptations were co-produced to ‘What’ materials with the learning collaborative, including updating key documents (shared by Region 1), gaining relevant governance approvals, and expanding NROL visibility with additional branding materials.

#### Recruit staff to deliver innovation, identify and prepare champions

Region 2 NROL Leads in collaboration with the learning collaborative, recruited NROL champions and group facilitators from high-readiness organisations. Innovation champions were key in supporting governance, advocacy, and troubleshooting. Group facilitators received targeted training, including technology and procedural sessions, and Region 1 observations. Meetings were held for delivery staff, champions, and leaders, with updates shared to wider teams.

### Step 3: Plan for and undertake piloting and evaluation of the adapted innovation

#### Ensure readiness

Innovation readiness was confirmed by the Region 2 NROL operational team and champions once the infrastructure, training, equipment, regional approvals and staffing were in place. Organisational readiness required local approvals, staff training and baseline service data to provide a reference point for evaluation.

#### Conduct small tests of change

Pilot testing was initially conducted at group-level through two pilot groups: a fatigue management group and a speech and language therapy group. These allowed assessment of acceptability and practicality. Staff and patient feedback informed iterative refinements to processes, logistics and group content.

#### Phased integration of interested organisations

Following successful group-level piloting, block-level piloting began. This involved phased integration of organisations with high-readiness into 6-week NROL blocks running seven groups (Cognitive education, Emotional adjustment, Dysarthria, Aphasia, Balance and Mobility, Upper limb, Community peer-support).

All interested organisations were integrated. Seven organisations (8 teams) adopted the programme initially, one further organisation (3 teams) was added in the second block and two more (2 teams) in the third. Five teams remained pending. The learning collaborative expanded to ensure regional representation.

Evaluation ran alongside implementation and centred on reviewing key peripheral components, using routinely collected clinical and service-delivery metrics and stakeholder feedback and was consistent with Region 1 evaluation. Region 2 NROL Leads co-produced summaries for stakeholders, including Region 1.

#### Document and classify responsive adaptations

Regular review meetings allowed iterative tailoring. Responsive adaptations are summarised in Figure 3 and included in the TIDieR-Rehab checklist (Supplemental file 2). Key responsive adaptations included tailoring to fit within the pathway such as supported inpatient participation and extended time in the community-based service when appropriate. Existing workforce influenced group content and facilitator roles (e.g. Allied health staff trained to lead well-being group). IT access issues prompted new resolution procedures, and breakout rooms (new functionality) were introduced, with procedures shared with Region 1.

### Step 4: Implement and maintain the adapted innovation

#### Conduct ongoing training

Findings from pilot testing and evaluation informed ongoing training. Staff were encouraged to observe live NROL sessions, in both regions, to support referral decisions and experiential learning. Cross-region observation fostered shared learning and networking. New staff were introduced to NROL as part of their service induction. New group facilitators received targeted training in both technology and procedures. General information about NROL was regularly disseminated by local and regional NROL staff and champions.

#### Consider sustainment

Sustainment was considered from the outset and involved continued dialogue with wide stakeholders, including decision-makers. Presentations were made to organisation transformation and improvement leaders. Advantages of the programme were identified and included staffing efficiencies, increased therapy contact, and savings in travel time, cost, and carbon emissions.

A co-produced blueprint outlined full implementation and evaluation plans. Further fixed-term project funding was secured, providing time to develop a business case for long-term regional investment. NROL delivery is ongoing.

#### Report adapted innovation described using TIDieR-Rehab checklist

The adapted NROL model for Region 2 was documented using the TIDieR-Rehab checklist, updated from Region 1’s version^13^ to reflect adaptations (see Supplemental file 2). This co-production process involved the Region 2 learning collaborative to ensure accuracy and resonance.

## Discussion

This study illustrates how the NROL telerehabilitation model was adapted for a new region using a structured, co-produced process. Core components were retained, while adaptations tailored role configuration, materials and fit within service pathways. The ADAPT guidance alongside implementation science knowledge provided a systematic approach to planning, piloting, implementing, and reporting the adapted model.

The adaptation process was initiated and led by Region 2 Stroke Rehab Leads who had relationships with local organisations and deep community knowledge facilitating implementation efforts. Involving the original region (Region 1) accelerated implementation by transferring tacit knowledge, tested materials, and credibility. While the ADAPT guidance warns working with innovation developers could risk fixed views or power imbalances,^21^ this did not materialise. The new region viewed the involvement positively, likely due to co-production principles, open dialogue, and mutual recognition. This approach provided a trusted environment to explore viable adaptations to meet local needs.

Creative solutions were fostered. Adjustments to role configurations and recruitment reflected the wider workforce landscape and leveraged diverse skill-sets. For example, Region 2 prioritised recruitment for two NROL technology support roles. The opportunity was harnessed to establish a patient-facing technology job creation and capitalised on dual skill-sets (e.g. assistant workforce) that could help sustain digital practice. Another example, in response to a system-wide lack of psychology staff, involved allied health staff being trained to deliver a well-being group with oversight from a clinical psychologist. This role agility aligns with the recent UK stroke guideline emphasis on what needs to be done rather than who does it.^9^ Creative solutions were also found for promoting NROL access, such as optimising fit within the service pathways (e.g. early exposure through inpatient participation) and introducing new materials (e.g. QR-coded keyring to share a patient information video).^38^ It may be that these adaptations actively enhanced joint decision-making between therapist and patient about service delivery preferences.^39^

Frameworks were applied selectively to help navigate the complexities of transferring across regions. The ADAPT guidance structured the adaptation process; CFIR and ISAT supported contextual analysis; and TIDieR-Rehab enabled transparent reporting. The process was non-linear, with evolving priorities. While these tools added rigour, they also introduced complexity. Overlapping constructs and terminology created challenges, requiring interpretive effort.^28,40^ Co-production, including clinical-academic involvement, helped translate conceptual guidance into practice-relevant actions.^33,41,42^ Insights are summarised in the “Key Considerations for Adaptation” box, which may be helpful for researchers and implementers seeking to scale telehealth models.

**Key considerations for adaptation**

• Embed co-production and shared learning: Engage stakeholders throughout.
• Retain core functions, use creative solutions to meet needs: Tailor staffing models, materials and fit within pathway.
• Use structured guidance flexibly: Apply frameworks to support planning and contextual fit.
• Anticipate complexity: Expect evolving roles and shifting priorities.
• Plan for sustainment early: Co-produce a blueprint and highlight efficiencies.

A limitation was that the project focused on a single adaptation case within well-connected national health system regions. Findings may not generalise to less resourced or less connected contexts. The use of embedded teams and qualitative feedback limits external validation but offers rich, practice-based insights. Further evaluation is needed to assess sustainability, cost-effectiveness, and equity. The stakeholder engagement activities and contributions presented (see Supplemental file 1) are illustrative rather than exhaustive; greater detail may have been valuable. It is also important to note that not all activities are tangible or easily captured. The relational aspects of co-production, though difficult to articulate, play an important role in implementation success,^39^ and warrant consideration in future work.

This paper highlights the complexity and effort required to adapt and sustainably scale interventions, a challenge often under-recognised in healthcare and funding agendas. It showed the value of working across silos and with the original region to share information, evaluation and know-how, including with decision-makers. Working as a cross-regional learning health system provided a means to accelerate the integration of NROL in the new context.^43^ The resource demands of thorough adaptation underscore the need for sustainable, bottom-up funding models. The results affirm that strategic adaptation must address clinician, patient, service, and system-level factors, with clinical-academic partnerships a potential mechanism for translating frameworks into scalable, context-sensitive tools.^44^

In addition to these system-level insights, the adaptation process highlighted that the NROL model of care may have potential application across other clinical and service contexts. As a delivery model, its components support principled adaptation across a range of contextual realities (e.g. different workforce configurations and practices). Its multidisciplinary, group-based approach brings together complementary therapies, contrasting with many existing stroke, neurological, and other allied health telerehabilitation interventions that are typically discipline-specific or individually delivered,^5–7,45^ offering an integrated and holistic approach with potential efficiencies. These features align with the needs of other multidisciplinary rehabilitation pathways, such as cardiac, frailty, and rheumatology. While promising, any adaptations should follow a structured process and include proportionate evaluation.

## Conclusion

This study offers for scale-up, providing a real-world example of adapting a complex telerehabilitation model using structured implementation science knowledge. It offers practical insights into how core components can be maintained while adapting elements to new contexts. As digital models expand, structured and context-sensitive adaptation grounded in co-production and implementation science approaches, will be essential for sustainable scale-up across diverse health systems. These findings may inform broader efforts in digital transformation, workforce redesign, and service innovation beyond telerehabilitation.

## Supplemental material

Supplemental Material - Embedding telerehabilitation in practice: Adapting NeuroRehabilitation OnLine (NROL) for a new context through co-production and implementation science knowledge Supplemental Material for Embedding telerehabilitation in practice: Adapting NeuroRehabilitation OnLine (NROL) for a new context through co-production and implementation science knowledge by Suzanne Ackerley, Amy Bastow, Ruth Witham, Louise Jackson, Adam Partington, Helen Vernon and Louise Connell in Digital Health.

Supplemental Material - Embedding telerehabilitation in practice: Adapting NeuroRehabilitation OnLine (NROL) for a new context through co-production and implementation science knowledge Supplemental Material for Embedding telerehabilitation in practice: Adapting NeuroRehabilitation OnLine (NROL) for a new context through co-production and implementation science knowledge by Suzanne Ackerley, Amy Bastow, Ruth Witham, Louise Jackson, Adam Partington, Helen Vernon and Louise Connell in Digital Health.
