# Supplementary material for: Embedding telerehabilitation in practice: Adapting NeuroRehabilitation OnLine (NROL) for a new context through co-production and implementation science knowledge
Source: Digit Health. 2026 Sep 18;12:20552076261484770. doi: 10.1177/20552076261484770 (PMC13724953; doi:10.1177/20552076261484770)
Supplement: Supplementary file 1 [file sj-pdf-1-dhj-10.1177_20552076261484770.pdf]

Supplementary file 1: Stakeholder Groups, Co-production Activities, and Contributions

Stakeholder group	Membership	Role	Examples of co-production activities*	Examples of contributions
NROL coalition	Region 2 Integrated Stroke Delivery Network (ISDN):<br>- Regional Stroke Rehab Leads from 10 Community/Early Supported Discharge teams led by a small team (n=5, Physiotherapists, Stroke Physicians, Programme Manager)	Assessed the need for innovation at Region 2, discussed candidate innovations, and considered NROL innovation-context fit. Designated project leadership.	Regional ISDN meetings (n=6)	Confirmed persistent challenges delivering recommended therapy but shared purpose to improve neurorehabilitation provision<br>Identified NROL as a candidate innovation, with alignment to stroke guidelines and service policy<br>Identified likely contextual barriers and facilitators for Region 2<br>Designated two Stroke Rehab Leads for project leadership
	Designated Region 2 NROL Leads:<br>- Regional Stroke Rehab Leads from the ISDN (n=2)	Initiated and provided project leadership	NROL project meetings (weekly)<br>Cross-regional NROL leads meetings (n=4)<br>Initial funding application (n=1) and associated meetings (n=6)	Established the cross-regional NROL coalition with Region 1<br>Synthesised contextual factors<br>Secured fixed-term seed funding<br>Determined adjustments to NROL operational team role configuration, recruited staff<br>Started relevant regional governance processes
	Designated Region 1 NROL Leads:<br>- NROL Operational lead (n=1)<br>- Clinical-Academic staff (n=2)	Provided advisory leadership to support Region 2 NROL project planning, with detailed knowledge of NROL and its implementation	Cross-regional NROL leads meetings (n=4)	Shared expertise, experience and resources, including contextual barriers and facilitators related to NROL at Region 1.<br>Integrated implementation science conceptual knowledge, with prior experience of ADAPT, TIDieR, and CFIR.<br>Synthesised contextual factors

Stakeholder group	Membership	Role	Examples of co-production activities*	Examples of contributions
NROL adaptation team	Region 2 NROL Designated Regional Stroke Leads (n=2)<br><br>Both regions dedicated NROL Operational Teams:<br>- Operational Leads (n=2)<br>- Technology Support staff (n=3)<br>- Assistants (n=1)<br>- Administrators (n=2)<br><br>Region 1 Clinical-academic project staff (n=2)	Led Region 2 NROL adaptation including training, piloting, evaluation, and consideration of sustainment.	NROL adaptation team meetings (n=10).<br><br>Information governance and IT discussions (as required)<br><br>Meetings with Region 2 service leads (as required)<br><br>Joint sessions between NROL operational team members (n=6), alongside observations of Region 1 NROL sessions with discussion (all groups observed)<br><br>Region 2 NROL champions meetings (monthly)<br><br>Region 2 NROL group meetings (~1/week during implementation, rotating groups)<br><br>NROL evaluation meetings (quarterly)<br><br>Policy and strategic discussions/presentations with Region 2 decision-makers (e.g. organisational transformation and improvement leaders and teams) (n=4)<br><br>Further funding application (n=1) and associated meetings (n = 8)<br><br>Co-authorship of NROL adaptation paper	Co-adapted NROL standard operating procedures and staff manual/documents<br><br>Co-adapted a NROL database. Developed a evaluation plan and reporting template. Co-produced evaluation summaries.<br><br>Gained regional governance approvals<br><br>Supported organisational readiness, including provision of equipment and materials, development of IT procedures for access, recruiting champions and guided baseline service data capture<br><br>Shared learning contributing to responsive adaptations (e.g. inpatient participation, breakout rooms)<br><br>Co-created patient videos to share experiences and views of NROL<br><br>Co-produced a blueprint of full implementation and evaluation plans<br><br>Secured further fixed-term project funding<br><br>Facilitated stakeholder-led advocacy and visibility of NROL<br><br>Ensured transparent reporting of the adaptation process, including a TIDieR description of NROL for Region 2
NROL learning collaborative, *iterated over* time	Region 2 existing workforce (across involved organisations):<br>- NROL champions (n=13),<br>- Team therapy staff (~25 group facilitators per NROL block)<br>- Service administrators (n=10)<br>- Service leads (n=9)<br>- Students (n=6)	Supported shared decision-making and co-production	Champions facilitated information governance and IT discussions at an organisational level (as required)<br><br>Pilot group feedback sessions (n=2), with therapy staff (n=3) and patients (n=6)<br><br>NROL feedback sessions (n=3), with therapy staff (n=18) and patients (n=24)<br><br>Observations of Region 1 and Region 2 NROL sessions, with discussions	Co-adapted NROL key documents<br><br>Gained local governance approvals<br><br>Supported organisational readiness, including recruiting champions and group facilitators<br><br>Shared expertise, experience, and feedback to inform implementation and refinement<br><br>Shaped responsive adaptations to group structure (e.g. decision-making on allied

Stakeholder group	Membership	Role	Examples of co-production activities*	Examples of contributions
NROL learning collaborative, *iterated over* time (CONTINUED)			Working group meetings, for NROL deliverers and operational team (weekly)<br>Regular wider service and professional meetings<br>Students co-delivered NROL sessions alongside therapy staff, with discussion and feedback	health staff being trained to deliver a well-being group with oversight from a clinical psychologist) and content<br>Facilitated stakeholder-led advocacy and visibility of NROL<br>Ensured accuracy and resonance of the TIDieR description of NROL for Region 2
	Region 2 patients (~30 participants per NROL block)	Supported shared decision-making and co-production	Pilot group feedback sessions (n=2), with therapy staff (n=3) and patients (n=6)<br>NROL feedback sessions (n=3), with therapy staff (n=18) and patients (n=24)<br>NROL patient videos (n=7)	Shaped responsive adaptations to group content to reflect patient needs and feedback (e.g. larger font on slides)<br>Co-created patient videos to share experiences and views of NROL
	Region 2 members of the stroke community:<br>- Stroke Association Support Coordinators (n=4)	Supported shared decision-making and co-production	Co-delivered NROL sessions alongside Region 2 therapy staff, with discussion and feedback	Shared expertise and resources to support group delivery, including provision of signposting and information on next steps after rehabilitation
	Region 1 Stroke and Neurorehabilitation Service Manager (n=1)	Supported shared decision-making and co-production	Strategic discussions with adaptation team (as required)<br>Co-authorship of adaptation paper	Shared business case development experience<br>Ensured transparent reporting of the adaptation process, including a TIDieR description of NROL for Region 2
	Region 1 NROL therapy staff (~40 group facilitators per NROL block)	Supported shared decision-making and co-production	Observations of Region 1 NROL sessions, with discussion	Shared expertise, experience, and feedback informing implementation and refinement of NROL at Region 2

**Note: Activities were undertaken collectively across stakeholder groups; not all individual members participated in every activity.*
