# Supplementary material for: Embedding telerehabilitation in practice: Adapting NeuroRehabilitation OnLine (NROL) for a new context through co-production and implementation science knowledge
Source: Digit Health. 2026 Sep 18;12:20552076261484770. doi: 10.1177/20552076261484770 (PMC13724953; doi:10.1177/20552076261484770)
Supplement: Supplementary file 2 [file sj-pdf-2-dhj-10.1177_20552076261484770.pdf]

Supplementary file 2: TIDieR-Rehab Checklist for NeuroRehabilitation OnLine (NROL) at Cheshire and Merseyside

The TIDieR-Rehab checklist[1] was used, informed by the Telehealth extension[2], to report the adapted NROL innovation at Cheshire and Merseyside (new region, Region 2).

Content was systematically updated from the TIDieR checklist described for Lancashire and South Cumbria (original region, Region 1)[3]:

1) The TIDieR checklist content for NROL at Lancashire and South Cumbria was reallocated into the sections of the TIDieR-Rehab checklist.
2) Each TIDieR-Rehab checklist section was updated to reflect components maintained and adapted with edits clearly marked to enable comparison:
   - **Bold font** for new/adapted content for NROL at Cheshire and Merseyside
   - ~~Strikethrough~~ for removed content
   - *Italics for additions/amendments* relevant to both regions

Supplementary Table 2 : TIDieR-Rehab Checklist for NeuroRehabilitation OnLine (NROL) at Cheshire and Merseyside

Item	Section	Explanation	Description
1	Brief name	Provide the name or a phrase that describes the intervention.	NeuroRehabilitation OnLine (NROL) **at Cheshire and Merseyside**
2	Why	Describe any rationale, theory or goal of the elements essential to the intervention.	NROL aims to enhance the rehabilitation offered for adult patients actively *receiving neurorehabilitation*. As part of a hybrid model of care, it utilises an online platform offering advantages to save time, energy and travel, enabling more therapy to be delivered using existing workforce.[4] Group therapy has favourable evidence,[5] leveraging the benefits of peer support.[6] By embedding NROL within the existing National Health Service (NHS) system, it supports sustainable service delivery. NROL aligns with strategic priorities, such as the use of data and digital technologies in healthcare.[7, 8] Collective use of the workforce, as a provider collaborative, fosters a community of practice and shared learning[9] *and helps support workforce capacity fluctuations*. NROL also allows for a critical mass of patients to receive group therapy where impairment incidence is low.
3	Who	Describe who the intervention is intended for.	Active community-based patients within stroke ~~and neurorehabilitation~~ services. This means patients are under an active episode of care at their local organisation and are part of a local treating therapist's caseload who is ultimately responsible for their overall rehabilitation and a key point of contact.
4	When	Describe when the intervention commenced in relation to the onset or stage of the condition and/or other relevant events.	Clinical reasoning should determine when NROL fits with a patient's overall rehabilitation. Patients are at varying chronicity.

5A and B	What	*Materials:* Describe any physical or informational materials used in the intervention, including those provided to participants or used in intervention delivery or in training of intervention providers. Provide information on where the materials can be accessed.<br><br>*AND*<br><br>*Procedures:* Describe each of the procedures, activities and/or processes used in the intervention, including any enabling or support activities	A secure 'NROL hub' collaboration platform (in Microsoft (MS) Teams) was created as a repository for shared resources *and group invites*. Key documents include an 'NROL standard operating procedure' and relevant approvals (e.g., Data Protection Impact Assessment). NROL branding (i.e., logo) increases visibility and facilitates team cohesion.<br><br>The materials and procedures for NROL referral, entry, delivery and exit phases are detailed below:<br><br>NROL referral materials and procedures:<br>Therapy team members identify, consent and refer suitable patients to appropriate NROL groups. They submit an 'NROL referral form' via the NROL hub, guided by an 'NROL staff manual' (*available on request*). An 'NROL patient information leaflet' is provided to patients.<br><br>NROL entry materials and procedures:<br>NROL team support staff process referrals and coordinate timetabling using an NROL database (MS Excel). Prior to programme start, an NROL staff member contacts new patients to complete 'NROL outcome measures'. Measures include the EQ-5D-5L for measuring health-related quality of life[10] and the patient-specific functional scale (PSFS)[11] for measuring activity performance *with modified wording for use in a neurological population,* and are administered through the patients' choice of either telephone or online questioning. Measures were chosen as they were consistent with existing data collection (e.g. **NROL data collection at the original region,** part of Stroke Sentinel National Audit Programme, UK), and had acceptable clinical utility according to the Tyson and Connell scale,[12] and measured domains identified within a NROL logic model as those the programme was aiming to influence. However, it is acknowledged that the measurement properties of these measures when obtained through telephone or online questioning is unknown, and the lack of outcome measures that have been tested psychometrically for remote delivery across neurological conditions is a challenge.[13] Additionally, NROL technology support staff members ensure that each patient has the necessary technology capability and equipment (hardware, software (MS Teams and email) and connectivity) to access NROL. An 'NROL technology support guide' is provided, *as necessary*. Patients participating in physical groups receive an 'NROL physical group guide' providing instructions on how to set up a safe exercise environment. Patients (and referrer) receive a personalised 'NROL entry email' that outlines their 6-week programme, and group invites.<br><br>NROL delivery materials and procedures:<br>Patients join NROL groups according to their personalised programme. These include targeted talking (e.g., cognition, communication, fatigue, well-being) and physical (e.g., balance/mobility and upper limb) therapy groups incorporating interactive, educational and practical elements. These groups are staffed by at least two group facilitators~~, often jointly by more than one discipline~~. Community groups are offered to all patients and include an NROL introduction session and an optional weekly peer support group.

			Throughout the programme and during each NROL session, technology assistance is available to both patients and staff. If a patient is expected to join a group but does not attend, the NROL technology support staff member makes contact to offer assistance. The maximum number of patients within a group is determined by the group facilitators to ensure the best experience for patients and staff.<br>NROL sessions are delivered by staff online (MS Teams) using existing devices equipped with webcams and microphones. For physical groups, a large-screen television is used for monitoring patients during exercise. The session content is developed by group facilitators based on evidence-based practice and may include discussions, demonstrations and presentations. The NROL database is used during sessions to access and record patient information, such as attendance. A telephone is required in case of adverse events. Group facilitators ensure that clinical notes are entered for all patients. Regular NROL staff meetings and group-specific meetings are held to discuss NROL delivery.<br>Patients participate in NROL using their agreed device. For patients in physical groups, a large screen device is necessary. Specific equipment, such as a frame or table, may be required to ensure stability. Patients are advised to wear comfortable footwear and clothing and have access to a suitable drink. Functional task practice may necessitate additional equipment, for example, pen and paper, kitchen items. Patients should have a telephone available in case of technology issues or adverse events. If patients have pendant alarms, they are encouraged to wear them.<br><br>NROL exit materials and procedures:<br>Patients (and referrer) receive an 'NROL exit email' that provides summary information. Outcome measures are repeated, and patients are asked to complete a satisfaction survey.<br><br>Evaluation:<br>Evaluation occurs concurrently with NROL delivery, **and consistent with NROL Lancashire and South Cumbria.**[4, 14] Service and patient-level data are sourced from the NROL database. The NROL leads undertake regular relevant analysis and summarise the data for feedback for multiple clinical and wider audiences.<br><br>Staff training:<br>NROL evaluation findings are discussed in NROL meetings as a basis for training. General NROL information is disseminated to staff across the participating trusts by NROL staff. Shared learning sessions**,** *including across regions*, are run to optimise knowledge translation. *Group facilitators receive one-on-one technology assistance.* New staff members are introduced to NROL during their orientation. Staff can observe NROL sessions for experiential learning.

6	Who provided	For each category of intervention provider (for example, psychologist, nursing assistant), describe their expertise, background and any specific training given.	Existing workforce:<br>NROL uses an existing NHS workforce alongside dedicated NROL roles for operations, administration and technology support.<br><br>NROL therapy staff:<br>Therapy staff (including nominated NROL champions and group facilitators) from ~~stroke and neurorehabilitation services~~ **community and/or early supported discharge stroke teams** (from integrated regional organisations) comprising Occupational therapists, Physiotherapists, Speech and Language therapists, Psychologists, *Dieticians*, ~~Pharmacists~~, Therapy assistants and Allied health ~~and psychology~~ students. **Ambition to include Pharmacists and Psychology students.**<br><br>NROL team support staff:<br>**Two dedicated NROL technology supports with dual skill-sets (Technology-therapy assistance, Technology-evaluation)**, NROL administrator, local organisation administrators.<br><br>Patient and public involvement:<br>Volunteers comprising ~~ex-NROL patients and~~ members of the stroke community as group facilitators **(e.g. Stroke Association Support Coordinators for peer-support discussion group). Ambition for Ex-NROL patients to be group facilitators. To date, patients are part of the regional NROL learning collaborative supporting co-production activity (e.g. review of group resource information, feedback for group content).**<br><br>Leadership:<br>Dedicated NROL operational lead and **Regional Stroke Rehab leads, with host organisation management sponsorship. Original region NROL leaders (Clinical-academic staff, NROL operational lead, host organisation Stroke and NeuroRehabilitation Service Manager) served in an advisory capacity.**
7	How	Describe the modes of delivery (such as face to face or by some other mechanism, such as internet or telephone of the intervention and whether it was provided individually or in a group.	Mode of delivery is online (MS Teams) and sessions are delivered *synchronously (i.e. in real-time)* by staff to groups of patients. This section overlaps with the 'What (material and procedures)', see Section 5.
8	Where	Describe the type(s) of environment(s) where the intervention occurred, including any necessary infrastructure or relevant features.	Group facilitators and the NROL technology support staff member**/s** attend from private, well-lit, and quiet workspaces at different geographical locations and organisations. Resourceful use of available workforce is encouraged, such as enabling staff to deliver NROL whilst working from home. A room with adequate space is required for demonstrating exercises for physical groups. Patients participate in NROL groups from their homes.
9	How much	Session duration:	Patients join NROL groups according to their personalised programme, with most sessions scheduled for 60 minutes.

		Specify the planned session(s) duration of the intervention	
		Essential elements amount: Specify the planned session(s) duration and/or repetition of the essential elements of the intervention.	*Active participation is promoted for approximately 50 minutes of the session, with about 5 minutes at the start of the session allowing for patients to join and 5 minutes at the end allowing for general discussion. Time-on-task has not been recorded for this group therapy. Patient participation can be dependent on level of engagement*.
		Frequency: Specify the planned frequency of the intervention	An NROL introductory session runs at the start of each block. Most targeted therapy groups run weekly.
		Intervention length: Specify the planned overall length of the intervention	NROL delivery is structured into recurring 6-week 'NROL' blocks to facilitate patient flow.
10	How challenging	Describe the approach(es) used to set and monitor the intervention/task challenge level.	The needs of patients in groups will be nuanced over time requiring a responsive approach. *Patients are encouraged to work at their challenge point. Staff monitor as relevant i.e. Clinical observation, discussion, measures (e.g. 'rating of perceived exertion').*
11	Regression/ Progression	Describe the planned regression and/or progression of dosage parameter(s), including when and how.	Patients can attend more than one NROL block if clinically indicated, providing they remain under the active care of their stroke ~~or neurorehabilitation~~ team.
12	Personalisation	Needs: If supplementary strategies were planned to enable the delivery of the essential elements of the intervention in response to specific individual or group needs, then describe what, why, when and how. Preferences: if the intervention was planned to be adapted for personal preferences, then describe what, why, when and how.	*Individualised 1:1 technology assistance as required*.<br>*Signposting to external agencies to support technology access*.
		Preferences: if the intervention was planned to be adapted for personal preferences, then describe what, why, when and how.	*Patients may join talking therapy or community groups with cameras off if discussed with NROL staff and considered appropriate, though this is discouraged. This does not apply to physical groups where camera use is mandatory*.
13	Protocol deviations	If there were deviations in the intervention protocol during the course of the study, describe the changes (what, why, when and how)	**Changes were made to when NROL was commenced relative to stroke onset. Staff were encouraged to offer inpatients the opportunity to trial NROL sessions with therapy staff support, to facilitate timely NROL referral during community-based stroke rehabilitation. In some services, the length of time in the active care of the team was extended to enable patients to complete their NROL programme**.<br><br>*MS Team Breakout rooms were introduced to enable individual support if required (e.g. technology issues, management of adverse events).*<br><br>Tailoring is required at patient, group and block levels. All referrals are screened by group facilitators to ensure session content is tailored. The structure of blocks, in terms of groups offered and frequency, actively considers

			patient need, workforce availability and capitalises on changing staff skill sets. **Session content, shared by Lancashire and South Cumbria, was tailored overtime. Minor variations in facilitator roles (e.g. Allied health staff trained to lead well-being group).**<br><br>NROL materials are continually edited to reflect updates. **Procedures implemented to enable rapid restoration of IT access.**
14	How well	Plan: If intervention adherence or fidelity was assessed, describe how and by whom, and if any strategies were used to maintain fidelity or improve fidelity, describe them.	Communications, resources and technology assistance are provided to optimise NROL entry and participation (see Section 5). Service data are obtained and reviewed to monitor performance.
		Actual: If intervention adherence or fidelity was assessed. Describe the extent to which the intervention was delivered as planned.	Implementation and evaluation of NROL in Cheshire and Mersey is ongoing and will be reported separately.
15	Harms	Plan: Describe the monitoring of adverse consequences.	Adverse consequences are recorded.
		Actual: Describe any adverse consequences, including the number, seriousness and relatedness to the interventions	Implementation and evaluation of NROL in Cheshire and Mersey is ongoing and will be reported separately.

References:

1. Signal N, Gomes E, Olsen S, et al. Enhancing the reporting quality of rehabilitation interventions through an extension of the Template for Intervention Description and Replication (TIDieR): the TIDieR-Rehab checklist and supplementary manual. *BMJ open* 2024; 14: e084320.
2. Rhon DI, Fritz JM, Kerns RD, et al. TIDieR-telehealth: precision in reporting of telehealth interventions used in clinical trials-unique considerations for the Template for the Intervention Description and Replication (TIDieR) checklist. *BMC medical research methodology* 2022; 22: 161.
3. Ackerley S, Wilson N, Boland P, et al. NeuroRehabilitation OnLine: Description of a regional multidisciplinary group telerehabilitation innovation for stroke and neurological conditions using the Template for Intervention Description and Replication checklist. *Digit Health* 2024; 10: 20552076241252263. 20240528. DOI: 10.1177/20552076241252263.
4. Ackerley S, Wilson N, Boland P, et al. Implementation of neurological group-based telerehabilitation within existing healthcare during the COVID-19 pandemic: a mixed methods evaluation. *BMC Health Serv Res* 2023; 23: 671. 20230621. DOI: 10.1186/s12913-023-09635-w.
5. Beare B, Doogan CE, Douglass-Kirk P, et al. Neuro-Rehabilitation OnLine (N-ROL): description and evaluation of a group-based telerehabilitation programme for acquired brain injury. *J Neurol Neurosurg Psychiatry* 2021; 92: 1354-1355. 20210623. DOI: 10.1136/jnnp-2021-326809.
6. Clark E, MacCrosain A, Ward NS, et al. The key features and role of peer support within group self-management interventions for stroke? A systematic review. *Disability and rehabilitation* 2020; 42: 307-316.

7. NHS England. 2022/23 priorities and operational planning guidance., https://www.england.nhs.uk/wp-content/uploads/2022/02/20211223-B1160-2022-23-priorities-and-operational-planning-guidance-v3.2.pdf (2022, accessed July 03,2024 2024).
8. 10 Year Health Plan for England: fit for the future, https://www.gov.uk/government/publications/10-year-health-plan-for-england-fit-for-the-future (2025, accessed August 8, 2025 2025).
9. National service model for an integrated community stroke service, https://www.england.nhs.uk/publication/national-service-model-for-an-integrated-community-stroke-service/ (2022, accessed August 8, 2025 2025).
10. Euroqol. EQ-5D-5L. , https://euroqol.org/information-and-support/euroqol-instruments/eq-5d-5l/.
11. Stratford P, Gill C, Westaway M, et al. Assessing disability and change on individual patients: a report of a patient specific measure. *Physiotherapy canada* 1995; 47: 258-263.
12. Tyson S and Connell L. The psychometric properties and clinical utility of measures of walking and mobility in neurological conditions: a systematic review. *Clinical rehabilitation* 2009; 23: 1018-1033.
13. Brito SAFd, Scianni AA, Peniche PdC, et al. Measurement properties of outcome measures used in neurological telerehabilitation: a systematic review protocol. *PloS one* 2022; 17: e0265841.
14. Ackerley S, Mason T, Partington A, et al. Can we enhance neurorehabilitation through regional implementation of group-based telerehabilitation? A mixed methods evaluation of NeuroRehabilitation OnLine (NROL). *medRxiv* 2025: 2025.2003.2010.25323664. DOI: 10.1101/2025.03.10.25323664.
